# The willingness of UK adults with intellectual disabilities to take COVID‐19 vaccines

**DOI:** 10.1111/jir.12884

**Published:** 2021-09-16

**Authors:** C. Hatton, T. Bailey, J. Bradshaw, S. Caton, S. Flynn, A. Gillooly, A. Jahoda, R. Maguire, A. Marriott, P. Mulhall, E. Oloidi, L. Taggart, S. Todd, D. Abbott, S. Beyer, N. Gore, P. Heslop, K. Scior, R. P. Hastings

**Affiliations:** ^1^ Department of Social Care and Social Work Manchester Metropolitan University Manchester UK; ^2^ Centre for Educational Development, Appraisal and Research University of Warwick Coventry UK; ^3^ Tizard Centre University of Kent Canterbury UK; ^4^ Institute of Health and Wellbeing University of Glasgow Glasgow UK; ^5^ National Development Team for Inclusion Bath UK; ^6^ Institute of Nursing and Health Research University of Ulster Jordanstown UK; ^7^ Unit for Development in Intellectual and Developmental Disabilities University of South Wales Pontypridd UK; ^8^ School for Policy Studies University of Bristol Bristol UK; ^9^ School of Medicine University of Cardiff Cardiff UK; ^10^ Division of Psychology and Language Sciences University College London London UK; ^11^ Centre for Developmental Psychiatry and Psychology Monash University Melbourne VIC Australia

**Keywords:** COVID‐19, intellectual disability, United Kingdom, vaccine willingness

## Abstract

**Background:**

Given the much greater COVID‐19 mortality risk experienced by people with intellectual disabilities (ID), understanding the willingness of people with ID to take a COVID‐19 vaccine is a major public health issue.

**Method:**

In December 2020 to February 2021, across the United Kingdom, 621 adults with ID were interviewed remotely and 348 family carers or support workers of adults with ID with greater needs completed an online survey, including a question on willingness to take a COVID‐19 vaccine if offered.

**Results:**

Eighty‐seven per cent of interviewees with ID were willing to take a COVID‐19 vaccine, with willingness associated with white ethnicity, having already had a flu vaccine, gaining information about COVID‐19 from television but not from social media, and knowing COVID‐19 social restrictions rules. A percentage of 81.7% of surveyed carers of adults with ID with greater needs reported that the person would be willing to take a COVID‐19 vaccine, with willingness associated with white ethnicity, having a health condition of concern in the context of COVID‐19, having had a flu vaccine, being close to someone who had died due to COVID‐19, and having shielded at some point during the pandemic.

**Conclusions:**

Reported willingness to take the COVID‐19 vaccine is high among adults with ID in the United Kingdom, with factors associated with willingness having clear implications for public health policy and practice.

## Introduction

Willingness to take vaccines (or in the negative – vaccine ‘hesitancy’) has long been a topic of research in public health and has become especially significant in the context of the current COVID‐19 pandemic, leading to multiple research studies internationally (for reviews, refer to Lin *et al*. 2020; Sallam 2021). Large within and across‐nation general population surveys of adults have been carried out since early in the COVID‐19 pandemic (i.e. from as early as March 2020). These research studies have generally found: considerable variability in willingness to accept a COVID‐19 vaccine across countries (Kerr *et al*. 2020; Lazarus *et al*. 2020); reducing willingness from the beginning of, or early in, the pandemic to the end of 2020 (Kerr *et al*. 2020; Lin *et al*. 2020); and that correlates of vaccine willingness/hesitancy vary across populations and countries (Lazarus *et al*. 2020).

People with intellectual disabilities (ID) have been shown to be at considerably increased risk for COVID‐19 infection and mortality following COVID‐19 infection compared with adults without ID (Clift *et al*. 2020; Gleason *et al*. 2021; Henderson *et al*. 2021; Williamson *et al*. 2021). The subpopulation of individuals with Down syndrome may be particularly vulnerable, potentially due to increased risks of immune dysregulation, congenital heart disease and respiratory conditions (Clift *et al*. 2020; Dard *et al*. 2020). Given such findings, and the underlying health inequities experienced by people with ID (Emerson and Hatton 2013), there have been calls internationally for people with ID to be prioritised for vaccines (e.g. Hotez *et al*. 2021). Countries including the United Kingdom (DHSC 2021) have acted in response to such calls, and the emerging data, to include people with ID in vaccine priority groups.

In this context, understanding vaccine willingness among populations of people with ID is crucial to inform national and potentially international public health policy, particularly as there is some evidence that people with ID can be fearful of a range of medical procedures, including needle phobia (Marriott 2017; Kupzyk and Allen 2019). In addition, there is not a tradition of vaccine hesitancy in developmental disabilities research, except notably to understand views about vaccines in parents of autistic children (e.g. Goin‐Kochel *et al*. 2020). As yet, there are also few data available about COVID‐19 vaccine willingness and people with ID. Lunsky *et al*. (2021) gathered data from 3371 workers in developmental disability services in Ontario, Canada, in early 2021 asking them about their own willingness to take the vaccine. The majority (62% very likely, a further 20% likely) reported that they would likely take the vaccine when offered. Given the importance of protecting individuals with ID, especially within residential settings (Landes *et al*. 2020), the Lunsky *et al*. study provided useful data to inform educational initiatives to increase vaccine uptake among paid care workers. Factors associated with non‐intent to have a COVID‐19 vaccine were: being younger, and endorsing beliefs about the lack of vaccine benefits, worry about rapid development of the vaccine, and concerns about side effects (Lunsky *et al*. 2021).

We could find only one study with any data from people with ID themselves. In a survey of family carers, paid staff, and people with intellectual and developmental disabilities in New York State, USA, Iadarola *et al*. (2021) reported data on vaccine willingness from 91 people with disabilities (including people with ID). ‘Vaccine intent’ was defined as definitely or probably being willing to have the vaccine and included a small number of people who had already had a vaccine, with 83.5% reporting positive vaccine intent. Correlates of vaccine intent were not examined separately for the group of disabled people responding directly to the survey.

Given variability in vaccine willingness across countries, variability in correlates of vaccine intent across countries, and the lack of data about vaccine willingness directly from or about individuals with ID, additional country‐specific data are needed urgently to inform vaccine policy. In the current study, we report data about willingness to take a COVID‐19 vaccine from:

1. adults with ID across the four UK countries (England, Northern Ireland, Scotland and Wales) who could take part in a remotely conducted interview (e.g. via videolink or telephone)
2. carers of adults with ID who were not able to take part in a direct interview with researchers.

For both samples, we report factors associated with willingness to take the COVID‐19 vaccine.

## Methods

### Participants

Two samples were recruited: adults with ID who were interviewed by a researcher (Cohort 1), and adults with ID who would not be able to take part in an interview, where family carers and support workers reported via an online survey (Cohort 2). Full details of the survey methods and participants can be found in Flynn *et al*. (2021).

Selected demographic information is presented in Table 1 for both cohorts. For Cohort 1, most of the participants were aged 16–44 years (68.2%), just over half were men (51.3%), the vast majority were white (94.7%), and 11% were people with Down syndrome. Substantial proportions of participants in Cohort 1 were living with their family (41.4%), living alone or with a partner (36.3%) or living with other people with ID in some form of supported housing or residential accommodation (22.3%). For Cohort 2, most people with ID were aged 16–45 (80.8%), a majority were men (56.5%), and the vast majority were white (94.1%). Almost half were people with ‘Profound and Multiple Learning (Intellectual) Disabilities’ (44.0%), and 18.3% were people with Down syndrome. Most adults with ID in Cohort 2 were living with their family (63.6%), relatively few people were living alone or with a partner (10.7%), and a quarter of people were living with other people with ID in some form of supported housing or residential accommodation (25.7%).

**Table 1:** Measures and participant data used for analyses reported here

Measure and coding	Cohort 1 (interviews with adults with intellectual disabilities	Cohort 2 (surveys with family carers and support workers of adults with intellectual disabilities) *n* (%)
Demographic variables		
Age		
16–44	*n* = 416 (68.2%)	*n* = 291 (80.8%)
45+	*n* = 194 (31.2%)	*n* = 69 (18.3%)
Gender		
Male	*n* = 312 (51.3%)	*n* = 204 (56.5%)
Female	*n* = 296 (48.7%)	*n* = 157 (43.5%)
Ethnicity		
White	White *n* = 588 (94.7%)	White *n* = 348 (94.1%)
All other groups	Other ethnic group *n* = 33 (5.3%)	Other ethnic group *n* = 22 (5.9%)
Person with Profound Multiple Learning Disabilities (PMLD)	*Data not collected*	
Yes		*n* = 164 (44.0%)
Not sure/No		*n* = 209 (56.0%)
Down syndrome		
Yes	*n* = 67 (11.0%)	*n* = 68 (18.3%)
No	*n* = 540 (89.0%)	*n* = 304 (81.7%)
Living situation		
Living with family	*n* = 247 (41.4%)	*n* = 213 (63.6%)
Living alone/with partner	*n* = 217 (36.3%)	*n* = 36 (10.7%)
Living with other people with intellectual disabilities	*n* = 133 (22.3%)	*n* = 86 (25.7%)
Health‐related variables		
Willingness to take the COVID‐19 vaccine if offered		
Yes	*n* = 474 (87.0%)	*n* = 267 (80.9%)
Not sure/No	*n* = 71 (13.0%)	*n* = 63 (19.1%)
Health condition that is a worry if the person gets coronavirus		
Yes	*n* = 315 (51.6%)	*n* = 245 (70.2%)
No	*n* = 295 (48.4%)	*n* = 104 (29.8%)
Self or carer rated health of the person today		
Good	*n* = 367 (60.1%)	*n* = 193 (53.0%)
OK	*n* = 205 (33.6%)	*n* = 133 (36.5%)
Not very good	*n* = 39 (6.4%)	*n* = 38 (10.4%)
Currently taking medications		
Yes	*n* = 477 (78.1%)	*n* = 290 (81.0%)
No	*n* = 134 (21.9%)	*n* = 68 (19.0%)
Has had flu vaccine in 2020/21 winter flu season		
Yes	*n* = 403 (66.3%)	*n* = 253 (72.5%)
No	*n* = 183 (30.1%)	*n* = 61 (17.5%)
Do not want a flu jab	*n* = 22 (3.6%)	*n* = 35 (10.0%)
Has shielded at any time since first lockdown		
Yes	*n* = 193 (31.3%)	*n* = 215 (59.6%)
No	*n* = 423 (68.7%)	*n* = 146 (40.4%)
Family member/friend/someone close to the person has died due to COVID‐19		
Yes	*n* = 98 (15.9%)	n = 29 (7.8%)
No	*n* = 499 (81.0%)	*n* = 327 (87.9%)
Information and knowledge of COVID‐19 rules		
Person's sources of information about COVID‐19 rules		*Data not collected*
Talking to friends/family	*n* = 274 (44.1%)	
Talking to support worker	*n* = 227 (36.6%)	
Television	*n* = 490 (78.9%)	
Radio	*n* = 96 (15.5%)	
Social media	*n* = 153 (24.6%)	
Newspapers	*n* = 39 (6.3%)	
Government website	*n* = 76 (12.2%)	
Phone news app	*n* = 86 (13.8%)	
Easy for person to find good information about COVID‐19		*Data not collected*
Yes	*n* = 427 (69.5%)	
No	*n* = 187 (30.5%)	
Person knows rules where the person lives about COVID‐19 restrictions		*Data not collected*
Yes	*n* = 521 (84.7%)	
Not sure/No	*n* = 187 (30.5%)	
Pandemic anxiety		
Pandemic Anxiety Scale (McElroy *et al*. 2020). Mean score (1 = Not at all worries; 2 = A little worried; 3 = Worried a lot) for 4 health‐related items: I am worried that I will catch coronavirus; I am worried that family and friends will catch coronavirus; I'm worried to leave my home right now; I'm worried I might give the infection to someone else. Excluded if 2 or more missing items from these 4.	Mean item score 1.99 (SD 0.61)	*Data not collected*

### Measures

Table 1 shows the set of measures used for the analyses in this paper, including how they were coded for the purposes of analysis. Responses reported by small numbers of participants (e.g. participants with a gender identity other than man or woman) were collapsed into broader categories or excluded from the specific analyses involving the relevant variable, but these participants were included in all other analyses (e.g. people identifying as a gender other than male or female were excluded from analyses concerning gender, but were included in all other analyses). An empty cell in Table 1 indicates that the particular measure was not collected with the particular cohort.

The selection and wording of measures was finalised through extensive consultation with groups of people with ID (particularly for Cohort 1 interviews) and family organisations (particularly for Cohort 2 surveys), to maximise relevance and accessibility. Where possible, survey items were adapted from those used in larger UK population surveys concerning people's experiences through the COVID‐19 pandemic, such as the Office for National Statistics Opinions and Lifestyle Survey (ONS 2021) and the Understanding Society COVID‐19 survey (Understanding Society 2021). A shortened version of the Pandemic Anxiety Scale (McElroy *et al*. 2020) was also used, retaining four of the seven items related to worries about COVID‐19 infection or leaving the house, and excluding worries about job and financial prospects.

For the analyses included here, measures included demographic factors, items related to health and COVID‐19, and (for Cohort 1) items concerning information and knowledge about COVID‐19 restrictions and anxiety related to the pandemic.

### Procedure

Recruitment of people into the study was facilitated through multiple methods across the United Kingdom, including through collaborating organisations in each country, social media, and wider networks of ID and family organisations across England, Northern Ireland, Scotland and Wales. Potential participants could express interest in the study via telephone, e‐mail, social media or clicking a link to the survey (for family carers and support staff only) on the research project website. Contact details of people who had indicated an interest in taking part in Cohort 1 were sent to research teams in each country, who contacted each person to talk through the project and send them the easy read participant information sheet. If people were still interested in taking part, at least 24 h later, the interviewer arranged to go through the consent process and, if the person consented, conduct the interview. For Cohort 2, the survey was available online, and included extensive participant information and consent questions before the survey started. No participants received an honorarium for participating.

For both cohorts, data were collected from mid‐December 2020 to the end of February 2021. Unconnected to this study, national announcements about all adults with ID being prioritised for COVID‐19 vaccines were made across the United Kingdom in the final week of February 2021.

For Cohort 1, trained research interviewers directly interviewed adults with ID via Zoom, telephone, Microsoft Teams, WhatsApp video call, Messenger video call or FaceTime, depending on the interviewee's preference. All interviewees had the capacity to take part in the interviews and gave their consent to do so before the interview was conducted. Data were entered directly into Qualtrics™ during the interviews by the interviewers. Three people preferred to self‐complete an online version of the survey, and in these cases, this was made available to them at their request. Participants were also able to have a supporter of their choice (e.g. family member and support staff) present at the interview. In all cases, flexibility was paramount to ensure that people with ID were able to participate in their preferred way. Interviews took typically 45 min to complete, and were usually completed in one sitting. Short breaks were offered during interviews when needed. All interviewers had experience of research interviewing and were trained via online training sessions within each country, with additional training sessions across all UK interviewers and regular supervision for interviewers. Of those who consented to be interviewed, 86% (*n* = 545) answered the question on vaccine hesitancy.

For Cohort 2, information was collected via an online Qualtrics™ survey about adults with ID who were not able to take part in an interview with a researcher. To gather data on this group, we surveyed their family carers or paid support staff. Eighty‐three per cent of participants were family carers of an adult with ID, and 15% of participants were paid support staff of an adult with ID. The remaining 3% of participants were other people who knew the adult with ID very well (e.g. a friend).

### Data analysis

Cohorts 1 and 2 data sets were analysed separately throughout, using SPSS 26. For categorical variables, potential associations between willingness to take a COVID‐19 vaccine were analysed using Fisher's exact test (for 2 × 2 tables) or chi‐square (for 3 × 2 tables), and relative risk (RR) (with 95% confidence intervals) for all comparisons. For mean scores on the four items taken from the Pandemic Anxiety Scale (McElroy *et al*. 2020), a *t*‐test (with Cohen's *d* to evaluate effect size) was conducted between those willing or unwilling to take the COVID‐19 vaccine. All tests were two‐sided or two‐tailed.

### Ethical approval

Research ethics approval was sought and obtained from the Manchester Metropolitan University Faculty of Health, Psychology and Social Care Faculty Research Ethics Committee.

### Data availability

A quantitative data set will be archived online in a form that will be available to researchers after all waves of data collection for the project have been completed.

## Results

### Cohort 1 – interviews with adults with intellectual disabilities

The vast majority of adults with ID interviewed (87.0%; *n* = 474) said that they would take a coronavirus vaccine if they were offered one, with 8.4% (*n* = 46) saying they were not sure, and 4.6% (*n* = 25) saying they would not take the vaccine.

Table 2 first presents potential associations between demographic factors and willingness to take a COVID‐19 vaccine. The only statistically significant association concerned ethnicity (Fisher's exact *P* = 0.033), with people in white ethnic groups 22% more likely (RR 1.22) to be willing to take a COVID‐19 vaccine if it was offered, although the 95% confidence interval for RR did contain 1, indicating that the two groups may not be distinct in their vaccine willingness.

**Table 2:** Factors potentially associated with willingness to take the COVID‐19 vaccine: Cohort 1

	Willingness to take COVID‐19 vaccine: Yes vs. Not Sure/No		
	*N* (%) Yes to COVID vaccine	Chi‐square/Fisher's exact test (two‐sided *P*)	Relative risk (95% CIs)
Demographic variables			
Age			
16–44	321 (85.6%)	*P* = 0.164	0.95 (0.89–1.01)
45+	149 (90.3%)		
Gender		*P* = 0.606	0.98 (0.92–1.05)
Man	233 (86.3%)		
Woman	236 (88.1%)		
Ethnicity		***P* = 0.033**	1.22 (0.95–1.56)
White	456 (87.7%)		
All other groups	18 (72.0%)		
Down syndrome		*P* = 0.531	0.96 (0.86–1.08)
Yes	48 (84.2%)		
No	421 (87.3%)		
Living situation			
Living with family	199 (85.4%)	Chi‐square = 0.601; df = 2; *P* = 0.740	RR Family vs. Others 0.98 (0.91–1.05)
Living alone/with partner	169 (87.1%)		RR Alone/Partner vs. Others 1.01 (0.94–1.08)
Living with other people with intellectual disabilities	91 (88.3%)		RR Other People with Intellectual disabilities vs. All Others 1.03 (0.47–1.51)
Health‐related variables			
Health condition that makes person worry about having coronavirus			
Yes	232 (86.2%)	*P* = 0.610	0.98 (0.92–1.05)
No	239 (87.9%)		
Self‐rated health			
Good	281 (86.2%)	*P* = 0.605	0.98 (0.92–1.05)
OK/Not very good	189 (87.9%)		
Currently taking medications			
Yes	367 (88.4%)	*P* = 0.070	1.08 (0.99–1.18)
No	104 (81.9%)		
Has had a flu vaccine in 20/21			
Yes	319 (92.2%)	***P* < 0.001**	**1.19 (1.1–1.3)**
No/Do not want a flu jab	150 (77.7%)		
Person has shielded			
Yes	149 (88.2%)	*P* = 0.680	1.02 (0.95–1.09)
No	325 (86.4%)		
Family member/friend has died due to COVID‐19			
Yes	71 (84.5%)	*P* = 0.477	0.97 (0.88–1.06)
No	388 (87.6%)		
Sources of information about coronavirus			
Talking to friends/family			
Yes	220 (86.6%)	*P* = 0.899	0.99 (0.93–1.06)
No	254 (87.3%)		
Talking to support worker			
Yes	168 (89.4%)	*P* = 0.284	1.04 (0.98–1.11)
No	306 (85.7%)		
Television			
Yes	388 (89.4%)	***P* = 0.002**	**1.15 (1.04–1.28)**
No	86 (77.5%)		
Radio			
Yes	78 (87.6%)	*P* = 1.000	1.01 (0.93–1.10)
No	396 (86.8%)		
Social media			
Yes	110 (79.1%)	***P* = 0.003**	**0.88 (0.81–0.97)**
No	364 (89.7%)		
Newspapers			
Yes	29 (93.5%)	*P* = 0.408	1.08 (0.98–1.19)
No	445 (86.6%)		
Government website			
Yes	64 (87.7%)	*P* = 1.000	1.01 (0.92–1.11)
No	410 (86.9%)		
Phone news app			
Yes	71 (87.7%)	*P* = 1.000	1.01 (0.92–1.10)
No	403 (86.9%)		
Information and rules about coronavirus			
Easy to find good information			
Yes	328 (87.7%)	*P* = 0.581	1.02 (0.95–1.10)
No	145 (85.8%)		
Knows social distancing rules			
Yes	410 (88.6%)	***P* = 0.013**	**1.14 (1.01–1.28)**
No	64 (78.0%)		
Pandemic anxiety (4 items scored 1 = Not at all worries; 2 = A little worried; 3 = Worried a lot)			
Pandemic anxiety	Yes to vaccine mean item score 1.98 (SD 0.62) Not Sure/No to vaccine mean item score 2.11 (SD 0.61)	***t* = −1.84; df = 533; *P* = 0.067**	Effect size Cohen's *d* = 0.25

Table 2 also presents potential associations between health‐related factors and willingness to take a COVID‐19 vaccine. There was a statistically significant association with people having had a flu vaccine in the 2020/21 winter flu season (Fisher's exact *P* < 0.001): people who had had a flu vaccine were 19% more likely (RR = 1.19) to be willing to take a COVID‐19 vaccine than people who had not or did not want a flu vaccine.

In terms of factors related to information and knowledge about coronavirus and social distancing rules, Table 2 reports that two information sources were associated with willingness to take a COVID‐19 vaccine. People who got information about coronavirus from the television were 15% more likely to be willing to take a COVID‐19 vaccine (Fisher's exact *P* = 0.002; RR = 1.15), and people who got information from social media were 12% less likely to be willing to take a COVID‐19 vaccine (Fisher's exact *P* = 0.003; RR = 0.88). People who said they knew the social distancing rules where they lived were also 14% more likely to be willing to take a COVID‐19 vaccine (Fisher's exact *P* = 0.013; RR = 1.14).

Finally, Table 2 shows there was no evidence of a difference in shortened Pandemic Anxiety Scale mean scores between those who would be willing to take a COVID‐19 vaccine and those who were not sure or would not be willing to take a vaccine (*t* = 1.84; df = 533; *P* = 0.067 (two‐tailed); Cohen's *d* = 0.25).

### Cohort 2 – survey of carers (including paid support staff) of adults with intellectual disabilities

The vast majority of carers of adults with ID surveyed reported that the person they care for would take a coronavirus vaccine if they were offered it (81.7%; *n* = 259), with 13.2% (*n* = 42) reporting they were not sure, and 5.0% (*n* = 16) reporting that the person would not take a vaccine.

Table 3 first presents potential associations between demographic factors and carer reports of the willingness of the person with ID to take a COVID‐19 vaccine. The only statistically significant association concerned ethnicity (Fisher's exact *P* = 0.043), with people in white ethnic groups reported to be 30% more likely (RR 1.30) to be willing to take a COVID‐19 vaccine if it was offered, although the 95% confidence interval for RR did contain 1.

**Table 3:** Factors potentially associated with willingness to take the COVID‐19 vaccine: Cohort 2

	Willingness to take COVID‐19 vaccine: Yes vs. Not Sure/No		
	*N* (%) Yes to COVID vaccine	Chi‐square/Fisher's exact test (two‐sided *P*)	Relative risk (95% CIs)
Demographic variables concerning the person with intellectual disabilities			
Age			
16–44	208 (81.3%)	*P* = 0.854	0.97 (0.86–1.10)
45+	51 (83.6%)		
Gender			
Man	152 (82.6%)	*P* = 0.662	1.03 (0.93–1.15)
Woman	109 (80.1%)		
Ethnicity			
White	252 (82.6%)	***P* = 0.043**	1.30 (0.94–1.79)
All other groups	14 (63.6%)		
PMLD			
Yes	119 (83.2%)	*P* = 0.398	1.05 (0.95–1.17)
Not sure/No	148 (79.1%)		
Down syndrome			
Yes	50 (87.7%)	*P* = 0.194	1.11 (0.99–1.24)
No	216 (79.4%)		
Living situation			
Living with family	158 (82.3%)	Chi‐square = 0.578; df = 2; *P* = 0.902	RR Family vs. Others 1.04 (0.92–1.17)
Living alone/with partner	26 (78.6%)		RR Alone/Partner vs. Others 0.97 (0.80–1.17)
Living with other people with intellectual disabilities	55 (79.7%)		RR Other People with Intellectual disabilities vs. All Others 0.98 (0.85–1.12)
Health‐related variables			
Health condition that carer worries about if the person gets coronavirus			
Yes	186 (85.3%)	***P* = 0.004**	**1.20 (1.04–1.39)**
No	66 (71.0%)		
Carer‐rated health of person			
Good	144 (80.9%)	*P* = 1.000	0.996 (0.90–1.11)
OK/Not very good	121 (81.2%)		
Currently taking medications			
Yes	207 (81.2%)	*P* = 0.467	0.94 (0.84–1.06)
No	56 (86.2%)		
Has had a flu vaccine in 20/21			
Yes	204 (91.1%)	***P* < 0.001**	**1.61 (1.34–1.93)**
No/Do not want a flu jab	51 (56.7%)		
Person has shielded			
Yes	163 (87.6%)	***P* = 0.001**	**1.20 (1.07–1.34)**
No	99 (73.3%)		
Someone close to person has died due to COVID‐19			
Yes	23 (100.0%)	***P* = 0.020**	**1.24 (1.17–1.31)**
No	238 (80.7%)		

Table 3 also presents potential associations between health‐related factors and willingness to take the COVID‐19 vaccine. There were four statistically significant associations.

People with ID were 20% more likely (RR 1.20) to be reported by carers as willing to take a COVID‐19 vaccine if the person had a health condition of concern if they caught COVID‐19, such as epilepsy (Fisher's exact *P* = 0.004). There was also a statistically significant association with people having had a flu vaccine in the 2020/21 winter flu season (Fisher's exact *P* < 0.001): people who had had a flu vaccine were 61% more likely (RR 1.61) to be reported to be willing to take a COVID‐19 vaccine than people who had not or were reported to not want a flu vaccine. People with ID were 20% more likely (RR 1.20) to be reported by carers as willing to take a COVID‐19 vaccine if the person had shielded at any point (Fisher's exact *P* = 0.001) during the pandemic up to the date of the survey. Finally, carer reporting of the person's willingness to take the COVID‐19 vaccine was 24% higher (RR 1.24) when carers reported that someone close to the person with ID had died of COVID‐19 (Fisher's exact test *P* = 0.020).

## Discussion

Direct comparisons with other UK vaccine willingness data are not possible given different timings of data collection and also variations in phrasing of questions. However, data derived from the Understanding Society UK panel survey reported 82% vaccine willingness in over 12 000 participants (Robertson *et al*. 2021); vaccine acceptance rates in UK samples repeated from May to October 2020 were consistently above 70% (Kerr *et al*. 2020); and in a sample of over 5000 UK adults vaccine acceptance was 71.7% in September to October 2020 (Freeman *et al*. 2020). All these were conducted with general population samples, and it was not reported if any data from people with ID were included.

These data suggest that acceptance of COVID‐19 vaccination (as reported in the current research) is at least as high in people with ID, if not slightly higher. Not only may this be good news given the additional risks of people with ID to serious consequences of COVID‐19 infection, but it indicates that people with ID are likely to play their part in COVID‐19 public health programmes. More carers were unsure about whether people with more significant impairments would be willing to have a vaccine (Cohort 2) than people with ID themselves (Cohort 1). However, this was mainly associated with a perception among Cohort 2 respondents that suitable supports and adjustments may not be in place to enable people with more severe/profound ID and multiple health needs to receive a vaccine (Flynn *et al*. 2021).

We did not observe associations between vaccine willingness and either age or gender; factors that have been examined extensively with inconsistent findings in other COVID‐19 vaccine willingness research (Freeman *et al*. 2020; Kerr *et al*. 2020; Lazarus *et al*. 2020; Robertson *et al*. 2021). We examined a number of other demographic factors that may be associated with vaccine willingness and found the same association in both cohorts: individuals from white ethnic groups were more willing to accept COVID‐19 vaccines compared with combined all other ethnic groups. These findings are similar to those in other UK samples (Freeman *et al*. 2020; Robertson *et al*. 2021). We did not have sufficient sample size to examine vaccine willingness in specific minority ethnic groups, but vaccine hesitancy is associated in particular with Black and Pakistani/Bangladeshi communities in other UK research (Robertson *et al*. 2021). More recent analysis of COVID‐19 vaccine coverage has found that, among non‐shielding adults with ID aged 16–64, vaccination rates are considerably lower across black, mixed, South Asian and other ethnic groups compared with white groups (OpenSafely 2021). Future research, therefore, should attempt to examine more fine‐grained analysis of any associations between ethnicity and vaccine willingness in adults with ID.

We found no evidence that adults with Down syndrome were more or less willing to take a COVID‐19 vaccine, and this is encouraging given that they have been identified as a particularly at risk group (Clift *et al*. 2020).

In the current study, health‐related factors were consistently associated with vaccine willingness in both cohorts of adults with ID. Vaccine willingness was associated with indicators of health *vulnerability* in Cohort 2 (having a health condition of concern in relation to COVID‐19, and having shielded at some point during the pandemic). Greater willingness in both cohorts associated with having received a flu vaccine in the 2020/21 winter season may indicate experience with vaccination is facilitative for adults with ID, or that again additional health vulnerability (and thus need to have a flu vaccine) is associated with vaccine willingness, or both explanations may be true. In Cohort 2 (but not Cohort 1), vaccine willingness was associated with the person being close to someone who had died of COVID‐19.

Finally, for Cohort 1 (questions were not asked for Cohort 2), getting information about COVID‐19 from television bulletins was associated with increased willingness, getting information from social media with lower levels of willingness, and reporting knowing about local pandemic related restrictions (i.e. being better informed) was associated with increased willingness.

This study has a number of limitations to be borne in mind when considering the findings. Participants identified themselves or people they were caring for or supporting as people with ID (or ‘learning disabilities’ in current UK parlance). The representativeness of the samples of participants is unknown (and there are no reliable data across the United Kingdom to make these comparisons). Although both samples were diverse in terms of gender, age, country and living situation, there were small numbers of people from any minority ethnic communities (5% in Cohort 1; 6% in Cohort 2). Reliable data on the ethnicity of adults with ID across the United Kingdom is surprisingly lacking; the 2011 Census for the population as a whole reported 12% of adults aged 18+ from minority ethnic communities in England and Wales (ONS 2018); 4% for adults aged 16+ in Scotland (National Records of Scotland n.d.) and 2% for people of all ages in Northern Ireland (Northern Ireland Statistics and Research Agency 2016).

As the data are cross‐sectional, we did not think it appropriate to build regression models examining the independent contribution of different correlates of vaccine willingness. This may be important in future. However, at this stage and to provide as much detail to policy makers and public health practitioners as possible, we focused on individual associations with appropriate effect size measures so that readers and organisations can independently assess the value of different correlates identified in the current study.

We suggest that these first data on vaccine willingness in adults with ID may be useful to inform public health efforts. In particular, subgroups less willing to accept COVID‐19 vaccines could be the focus of educational interventions especially if these can be informed by additional research to understand hesitancy in some sub‐groups. Given that adults with ID reported using television broadcasts as a main source of information about COVID‐19 (Flynn *et al*. 2021) and that accessing information from the television was also associated with increased vaccine willingness, vaccine information targeted at (or made appropriately accessible for) adults with ID via television broadcasts may be particularly useful, compared with the current focus on producing easier‐to‐read web resources. In terms of adjustments that should be made in vaccine programmes, our findings also suggest that experience with vaccines (or perhaps other preparation to increase familiarity with/reduce anxiety about the vaccination procedure) may be crucial for some adults with ID – making reasonable adjustments at all stages to improve the likelihood of people being vaccinated is likely to be crucial in maximising vaccination rates (Rotenberg *et al*. 2021).

Given the variation in COVID‐19 vaccine willingness around the world, variability in predictors of willingness in different countries, and the lack of data on vaccine willingness among people with ID, more research, allied to urgent action, is clearly needed.

## Source of funding

This research (grant COV0196) was funded by UK Research and Innovation (Medical Research Council) and supported by the Department for Health and Social Care (National Institute for Health Research) as part of the UKRI‐DHSC COVID‐19 Rapid Response Rolling Call. The views expressed in this publication are those of the authors and not necessarily those of DHSC, NIHR, UKRI or MRC.

## Conflicts of interest

The authors have not identified any conflicts of interest.

## Data Availability

A quantitative data set will be archived online in a form that will be available to researchers after all waves of data collection for the project have been completed.
